# Real-World Retrospective Report on the Efficacy, Tolerability, and Molecular Responses to Ropeginterferon-α2b in Patients with Myeloproliferative Neoplasms

**DOI:** 10.3390/jcm15010128

**Published:** 2025-12-24

**Authors:** Matthias Christen, Domenic Kaderli, Milos Ratknic, Adrián Dante de Angelis, Philipp Stefan Aebi, Naomi Porret, Joëlle Tchinda, Natalia Baran, Wuddri Rim, Pascale Julia Tanner, Sebastian Mathes, Anne Angelillo-Scherrer, Alicia Rovó, Sara C. Meyer

**Affiliations:** 1 Department of Hematology and Central Hematology Laboratory, Inselspital, Bern University Hospital, University of Bern, 3010 Bern, Switzerlandnaomi.porret@insel.ch (N.P.); sebastian.mathes@insel.ch (S.M.); anne.angelillo-scherrer@insel.ch (A.A.-S.);; 2 Department of Biomedical Research, University of Bern, 3008 Bern, Switzerland

**Keywords:** polycythemia vera, essential thrombocytopenia, primary myelofibrosis, MPN-U, JAK2 V617F, pegylated interferon alpha

## Abstract

**Background:** Ropeginterferon alfa-2b (Ropeg-IFNa) is increasingly used in myeloproliferative neoplasms (MPN), particularly polycythemia vera, but real-world data across subtypes are limited. We evaluated clinical and molecular responses to Ropeg-IFNa in routine practice. **Methods:** We retrospectively analyzed 20 JAK2^V617F^-positive MPN patients treated at a tertiary center. Baseline features, dosing, treatment line, hematologic responses, adverse events, and serial JAK2^V617F^ variant allele frequency (VAF) were extracted from records. **Results:** Median age at initiation was 53 years; 55% were ELN high-risk. Ropeg-IFNa was started first-line or after peginterferon alfa-2a, hydroxyurea, or a tapered JAK2 inhibitor. Mean treatment duration was 14 ± 11 months at 195 ± 143 µg Q2W. Hematologic control increased from 45% at the start to 60% at the last follow-up. Among patients with serial molecular monitoring (n = 11), median JAK2^V617F^ VAF declined from 21.2 to 12.7%. Ropeg-IFNa was generally well tolerated; adverse effects were mostly manageable, although 3/20 (15%) discontinued due to side effects, including mood disturbances, while others continued with supportive care and dose adjustments. **Conclusions:** In this single-center cohort, Ropeg-IFNa was tolerable and associated with improved hematologic control and modest VAF reductions, supporting its use in multi-subtype MPN cohorts. These findings underscore the value of longitudinal driver-mutation monitoring during therapy.

## 1. Introduction

Myeloproliferative neoplasms (MPNs), including polycythemia vera (PV), essential thrombocythemia (ET), prefibrotic primary myelofibrosis (pre-PMF), and unclassifiable MPN (MPN-U), are myeloid neoplasms marked by the overproduction of mature myeloid blood cells including erythrocytes, platelets and/or leukocytes [1]. Most cases are driven by the somatic JAK2 V617F variant, causing constitutive JAK-STAT signaling and myeloid hyperproliferation [2,3]. Standard care aims to control cytoses to prevent thrombosis and to alleviate splenomegaly and symptoms [4,5]. The disease can progress to myelofibrosis, and risks of leukemic transformation vary by entity [6].

Unlike purely cytoreductive therapies, interferon-alpha (IFNa) normalizes counts and reduces the JAK2 V617F burden, indicating disease-modifying potential [7]. Pegylated IFNa, such as peginterferon alfa-2a, has seen growing use due to extended half-life and improved practicality [7]. Ropeginterferon alfa-2b (Ropeg-IFNa) is a third-generation, monopegylated, long-acting IFNa administered subcutaneously every two weeks. Randomized trials showed non-inferior control of cytoses versus hydroxyurea/best available therapy and higher rates of complete hematologic response with deeper molecular remissions, suggesting superior long-term outcomes in PV [8,9,10]. Consequently, Ropeg-IFNa is increasingly adopted, particularly in younger patients or where disease modification is prioritized [11,12].

Because trial populations may not fully reflect routine practice, real-world data are essential. Several small series outside trials report favorable outcomes: initial experience in nine patients showed ~63% hematologic and molecular responses with mostly mild toxicity and no discontinuations for intolerance [13]. Subsequent cohorts, including prior peginterferon-exposed ET/PV, found Ropeg-IFNa well tolerated with high response rates [14,15]. An Italian single-center PV study confirmed effective cytosis control and early molecular responses, often at lower doses and sometimes combined with hydroxyurea [16]. These reports highlight local practice patterns and the need for broader real-world evidence [13,14,15,16].

Ropeg-IFNa was approved for PV in the EU (February 2019), Switzerland (July 2020), and the United States (November 2021). Pegylated IFNa has long been used in ET [17,18], a phase 3 trial of Ropeg-IFNa versus anagrelide in ET recently reported positive results [19], and pegylated IFNa is increasingly used in pre-PMF with multiple reports published [20]. Notably, recent shortages of other pegylated IFNa preparations have prompted switching to Ropeg-IFNa, with practical guidance available [21]. We therefore conducted a retrospective, real-world analysis of efficacy, tolerability, and molecular responses to Ropeg-IFNa in JAK2 V617F-mutant MPNs including PV, ET, pre-PMF, and MPN-U treated at our tertiary center.

## 2. Materials and Methods

*Study design and patients:* We conducted a single-center, retrospective observational study of Ropeg-IFNa in JAK2 V617F-mutant MPN. All patients initiating Ropeg-IFNa at our institution from Swiss approval in July 2020 to January 2025 were screened. Eligible entities included PV and non-PV MPN (ET, pre-PMF, MPN-U). Non-PV cases were treated off-label. “Baseline” was defined as the date of Ropeg-IFNa initiation. Exclusion criteria were the absence of confirmed JAK2 V617F variant and, for patients remaining on therapy (i.e., without early discontinuation), <3 months exposure. Patients were identified from hospital records and routine MPN clinic visits. A chart review initially identified 26 patients. Six lacked written research consent and were excluded, leaving twenty patients followed from MPN diagnosis to last visit (April 2025).

*Ethics*: The study adhered to the Declaration of Helsinki and Swiss human research regulations. All participants provided written general consent, and the study was assessed by the local ethics committee (KEK Bern, ID 2025-01132).

*Data collection:* Clinical data were extracted from routine records. Baseline variables included age, gender, MPN subtype, prior MPN therapies, and indication for starting Ropeg-IFNa. Peripheral blood counts, including hemoglobin, hematocrit, leukocytes, and platelets, were captured longitudinally where available and specifically worked-up at predefined timepoints: diagnosis (Dgn), Ropeg-IFNa start (Start), ~3 months on therapy (Cont), and last follow-up (Last; included if >3 months from Start). Hematologic response followed ELN criteria [22,23]: hematocrit ≤ 45%, leukocytes ≤ 10 G/L, platelets ≤ 400 G/L. Phlebotomy frequency and spleen size were inconsistently available and not analyzed. Molecular response was assessed by serial peripheral-blood JAK2 V617F VAF using ddPCR in our clinical molecular genetics laboratory; limit of detection 0.05% VAF. JAK2 V617F VAF was recorded at first available measurement, at Ropeg-IFNa initiation (or up to 6 months before if not available), and all subsequent available time points. Serial testing was performed when clinically available. Targeted myeloid genomics, when clinically indicated, used the TSO500 65-gene NGS panel (Illumina, Inc., San Diego, CA, USA). Symptom burden was evaluated by the validated MPN-10 Symptom Assessment Form (MPN10 SAF; 0–100 total score) at diagnosis, pre-treatment, and, when available, on-treatment [24]. Tolerability and safety data were extracted from charts, including adverse events, laboratory abnormalities, dose modifications/interruptions, and discontinuations with reasons (e.g., tolerability, insufficient efficacy).

*Dosing outcomes and status categories:* A stable maintenance dose was defined as a dose unchanged for ≥3 consecutive visits or across ≥2 visits >60 days apart with no subsequent increase. At last follow-up, patients were categorized as ongoing or discontinued Ropeg-IFNa.

*Statistical analysis:* Analyses used *GraphPad Prism* v8.0.1 (GraphPad Software, LLC, Boston, MA, USA) and *R* v4.5.0 (GraphPad Software, LLC, Boston, MA, USA). Descriptive statistics summarized characteristics and outcomes: continuous variables as median (range) or mean ± SD, and categorical as counts and percentages. Shapiro–Wilk, Anderson–Darling, Kolmogorov–Smirnov, and D’Agostino and Pearson tests assessed normality of paired differences. Paired tests (paired *t*-test or Wilcoxon signed-rank) compared blood counts, JAK2 V617F VAF, and MPN10 TSS over time. Two-sided *p* < 0.05 was considered significant. Given the small sample, analyses were exploratory, emphasizing descriptive trends in hematologic/molecular responses and qualitative tolerability.

## 3. Results

### 3.1. Patient Characteristics at Ropeg-IFNa Initiation

We included 20 patients with JAK2 V617F-mutant MPN treated with Ropeg-IFNa since July 2020. Nine were non-PV patients including ET, pre-PMF and MPN-U. Median age at baseline was 52.9 years (range 34–77, 55% male, Table 1). The median interval from MPN diagnosis to Ropeg-IFNa start was 1.8 years (2 months–25 years). Eleven patients (55%) had a high-risk profile for thromboembolic complications according to ELN criteria (age > 60 or prior thrombosis), ten of which had a history of thromboembolic events. Cytoreductive treatments prior to Ropeg-IFNa (Table 1) included hydroxyurea (n = 13), peginterferon alfa-2a (n = 8, PV only), and ruxolitinib (n = 2, one PV, one MPN-U). Over the course of their prior treatment, fourteen patients received one line of cytoreductive therapy, three received two sequential lines, and one patient was exposed to three lines before starting Ropeg-IFNa. Phlebotomy had been performed in 8/11 PV and one MPN-U patient. Antiplatelet/anticoagulant therapy was administered in 18/20 (90%).

Baseline blood counts were generally within upper normal ranges (Table 1) consistent with prior cytoreductive treatment. PV patients were mostly within ELN target values [22], with 4/11 patients surpassing target values for hematocrit, platelets, or both. Mean platelet count remained above the target range in ET and pre-PMF. Leukocyte control was less complete in pre-PMF and MPN-U (values near the 10 G/L threshold) than in PV/ET. All patients carried JAK2 V617F and no exon 12 mutations were detected. Quantitative variant allele frequency (VAF) data were available in 13/20 (65%), with a median of 12.0%. JAK2 V617F VAFs were higher in PV (21.2%) and pre-PMF (37.0%) than in ET (9.1%) and MPN-U (12.4%) (Table 1). A myeloid NGS panel was performed in 9/20 (45%). Additional mutations were found in 5/9 (56%), including *ASXL1* (n = 2; one PV, one ET) and single occurrences of *EZH2*, *DNMT3A*, *TET2*, *BCOR*, and *NF1* mutations (across PV, ET, MPN-U). MPN-SAF was available for 12/20 (60%), and the baseline median total symptom score was 5.5/100.

### 3.2. Characteristics of Ropeg-IFNa Administration

At baseline, 15 patients (75%) were on active MPN-directed treatment beyond phlebotomy, anticoagulation or antiplatelet therapy: peginterferon alfa-2a (n = 7), hydroxyurea (n = 6), combined peginterferon + hydroxyurea (n = 1), or ruxolitinib (n = 1). The remaining five received phlebotomy only (n = 3) or antithrombotic prophylaxis only (n = 2). Peginterferon was discontinued one week before the first Ropeg-IFNa dose. Hydroxyurea was generally tapered over weeks (one abrupt stop). Ruxolitinib overlapped for two weeks and was tapered. Phlebotomy continued temporarily after Ropeg-IFNa start in five patients (4 PV, 1 MPN-U). Anticoagulant/antiplatelet therapy was continued unchanged. Reasons for switching to or starting Ropeg-IFNa fell into three categories: incomplete efficacy of previous therapy (9/20, 45%; hematologic control n = 7, symptom control n = 1, clonal suppression n = 1), tolerability/long-term safety concerns with previous therapy (7/20, 35%), and ease in use/availability (4/20, 20%; e.g., reduced injection frequency or better access). Among PV patients, 8/11 switched directly from peginterferon alfa-2a to Ropeg-IFNa, most commonly for ease in use/availability (4/8) or insufficient hematologic control (3/8). One patient who had developed depressive symptoms on peginterferon alfa-2a experienced recurrent mood disturbance on Ropeg-IFNa and discontinued treatment.

**Figure 1:**
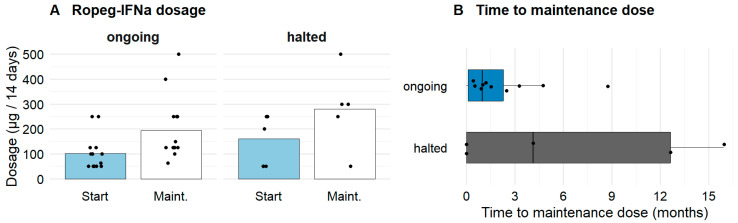
**Ropeg-IFNa dosing characteristics.** (**A**) Starting and maintenance dosages of Ropeg-IFNa, stratified for treatment continuation (ongoing vs. halted). Patients with ongoing Ropeg-IFNa therapy were enriched for lower starting dosages and lower maintenance dosages. Bars show group means with individual values overlaid. (**B**) Time to maintenance dosage (defined as ≥3 visits at stable dose or >60 days at stable dose between two visits) separated by sustainability of treatment in patients with continued Ropeg-IFNa vs. patients, who have halted Ropeg-IFNa treatment.

Ropeg-IFNa was first used from September 2021 at 250 µg Q2W. Among the first ten patients (to August 2023), th mean starting dose was 166 ± 78 µg Q2W; in the subsequent ten (September 2023 to January 2025) it was 60 ± 20 µg Q2W, consistent with evolving dose-titration practice and earlier recognition of side effects at higher starts. By subtype, starting doses were highest in PV (155.7 ± 85.7 µg Q2W) and lowest in MPN-U (62.5 ± 25 µg Q2W). Median treatment duration was 11.5 (1.9–41.2) months. Nineteen of twenty patients reached a stable maintenance dose. One was still in titration at the last follow-up. Of those with stable ongoing treatment (n = 14), mean starting and maintenance doses were 100.9 ± 69.4 µg and 193.8 ± 124.7 µg Q2W, respectively (Figure 1). In patients who discontinued (n = 5), starting and maintenance doses were 160 ± 102.5 µg and 280 ± 160.5 µg Q2W. Time to maintenance was shorter in ongoing vs. discontinued patients (1.8 ± 2.4 vs. 6.5 ± 7.4 months). Differences were not statistically significant but suggest higher/slower escalation in those who stopped therapy.

### 3.3. Hematologic and Molecular Responses to Ropeg-IFNa

**Figure 2:**
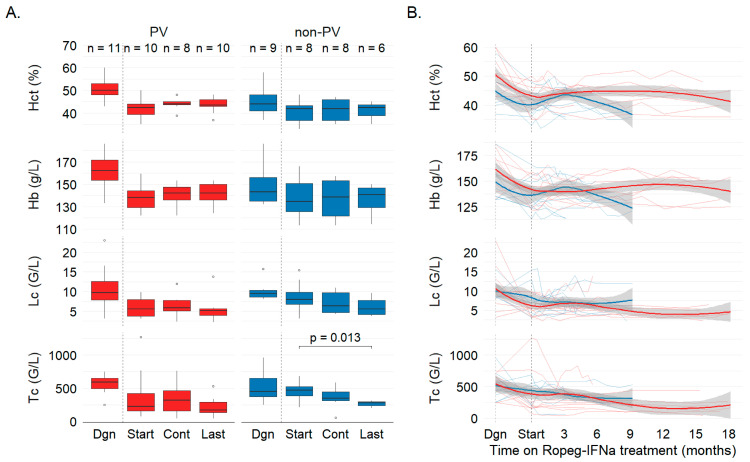
**Hematologic response as reflected by peripheral blood counts in MPN patients on Ropeg-IFNa treatment.** (**A**) Peripheral blood counts at diagnosis of MPN (Dgn), initiation of Ropeg-IFNa treatment (Start), upon continued therapy for approx. 3 months (Cont) and, at last, follow-up (Last) are indicated for polycythemia vera (PV, red) and non-polycythemia vera (non-PV, blue) MPN subtypes. Boxplots show values for hematocrit (Hct, %), hemoglobin (Hb, g/L), leukocytes (Lc, G/L), and thrombocytes (Tc, G/L). Each box represents the distribution of one parameter per group and time point with sample sizes (n) indicated in the top row. Statistical comparisons of values at start of Ropeg-IFNa (start) vs. subsequent time points were performed by paired Wilcoxon tests. *p*-values are indicated if significance was reached (*p* < 0.05). (**B**) Longitudinal trends in hematological parameters. Lines represent individual patients; bold curves indicate smoothed LOESS trends with 95% CI (gray). The trend line and individual trajectories are truncated at the last time point with at least two patients contributing data.

Hematologic efficacy was evaluated per ELN criteria at predefined time points (Dgn, Start, Cont, Last; Figure 2). At Start, 9/20 (45%) met hematologic response: 6/11 PV (55%) and 3/9 non-PV (33%), reflecting prior cytoreductive therapy. Under Ropeg-IFNa, non-PV platelet counts decreased from 458 ± 140 to 265 ± 46.8 G/L (*p* = 0.013), while hematocrit and leukocytes were maintained or modestly reduced. By Last, responders rose to 12/20 (60%): 6/11 PV (55%) and 6/9 non-PV (67%), primarily via improved control of thrombocytosis and leukocytosis. Of seven patients who started Ropeg-IFNa to improve hematologic control, five improved and continued, while two did not and discontinued.

**Figure 3:**
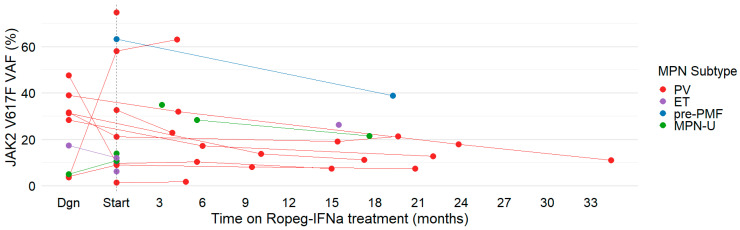
**JAK2 V617F VAF monitoring over time.** JAK2 V617F VAF was plotted for each patient, regardless of duration of follow-up assessments. Colors indicate MPN subtype. The dashed vertical line denotes the initiation of Ropeg-IFNa treatment (Start). Measurements up to 6 months before initiation of Ropeg-IFNa treatment were considered as JAK2 V617F value at start.

Molecular response (JAK2 V617F VAF) was analyzed where available (Figure 3). At Start, 13/20 (65%) had VAF data (range 1.4–74.6%, median 12.0%). Five also had sequential pre-Ropeg-IFNa measures (VAF rose in three, fell in two under prior therapy). During Ropeg-IFNa, eleven patients had serial VAFs enabling longitudinal analysis (seven beginning at Start; four beginning before or after Start). Among these 11 patients, median VAF declined from 21.2% to 12.7% over 14.8 (range 3.8–29.7) months. Given borderline normality of paired difference, VAF was estimated with paired two-tailed *t*-test (*p* = 0.0495), as well as Wilcoxon signed rank test (*p* = 0.042). The median paired change was −2.53 percentage points, consistent with the observed trend in decreasing VAF. No complete molecular remissions were observed. Reductions tended to be greater with higher baseline VAF (>20%). Most decreases occurred in PV, with a similar reduction in one MPN-U. The largest drop (>20% absolute) occurred in a pre-PMF patient over 20 months. ET patients had no sequential on-treatment VAF monitoring available, owing to local practice patterns during the study period and generally low baseline VAFs in ET, so longitudinal molecular inference in ET was not possible.

### 3.4. Side Effects of Ropeg-IFNa Treatment in MPN

**Figure 4:**
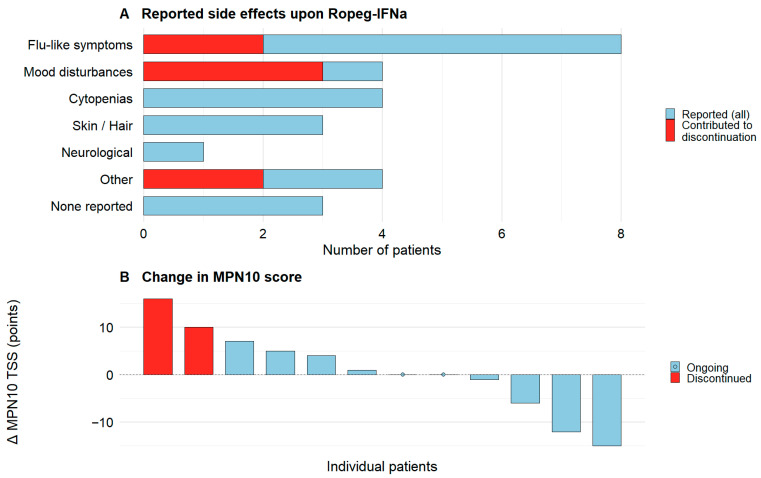
**Tolerability of Ropeg-IFNa.** (**A**) Number of patients with reported side effects during Ropeg-IFNa treatment (blue bars), with side effects contributing to therapy discontinuation in three patients as red bars. (**B**) Waterfall plot illustrating the changes in MPN10 total symptom score (TSS) from baseline to last available measurement in the 12 patients with available sequential MPN10 TSS measurements. Patients who discontinued therapy are indicated in red and patients who continued therapy are indicated in blue. All patients who discontinued Ropeg-IFNa were switched to alternative therapy (hydroxyurea or JAK2 inhibition, see Results section).

Treatment-emergent side effects occurred in 17/20 patients (85%, Figure 4A), and were mostly mild and manageable. All adverse events were grade 1–2 according to CTCAE v5.0, including events leading to treatment discontinuation, and no treatment-related hospitalizations occurred. Flu-like symptoms, including fever, fatigue, headache, malaise, and myalgias/arthralgias, were most common (8/20). Mood disturbances such as depression, anxiety, mood swings/inner restlessness, and cytopenias (predominantly thrombocytopenia or neutropenia) each occurred in 4/20. Additional events included dermatologic (itching, hair loss), neurologic (migraine with visual disturbance), and other (Raynaud’s phenomenon, hypertension, loss of appetite). Among patients who continued therapy, adverse effects were generally controlled with supportive measures (e.g., antipyretics/analgesics) and dose adjustments. Among patients with paired MPN10 assessments at Ropeg-IFNa initiation and last available measurement (n = 12), there was no statistically significant increase or decrease in symptom burden as reflected by MPN10 TSS (paired two-tailed t test, *p* = 0.77). Two patients that discontinued treatment because of side effects showed the biggest increase in MPN10 TSS at the last available measurement (Figure 4B), while scores showed great variability in ten patients with ongoing treatment.

Ropeg-IFNa was discontinued in 5/20 patients (25%): 2 for insufficient response and 3 for side effects (all CTCAE grade 1–2, no hospitalizations). The two non-responders stopped after 6.7 and 15.4 months at 250 and 500 µg Q2W, respectively. Specifically, they discontinued for inadequate hematocrit control. Among those discontinuing for toxicity, one patient experienced restlessness, nausea, shortness of breath, gum pain, and joint pain within four days after each injection, together with a persistent depressive mood. A second patient, who had previously developed depression on peginterferon alfa-2a, noted a transient improvement after switching therapy but had a recurrence of mood symptoms once on Ropeg-IFNa. The third patient reported nausea, mild diarrhea, fatigue, headaches, and progressively worsening mood changes. Discontinuations due to tolerability occurred at a median of 5.6 months (range 1.9–22.0), at doses of 300 µg Q2W in two cases and 50 µg Q2W in one. After stopping Ropeg-IFNa, all five patients transitioned to hydroxyurea (n = 3) or JAK2 inhibitors (n = 2), with one of the latter also receiving phlebotomies.

## 4. Discussion

Real-world data for Ropeg-IFNa across MPN subtypes remain sparse. We add to this evidence by reporting efficacy, tolerability, and molecular outcomes at our tertiary center since approval. Hematologic control was maintained or improved relative to antecedent therapy: 45% met ELN response at initiation, rising to 60% at last follow-up after ~14 months on Ropeg-IFNa. These rates accord with prior real-world series (57–67%) [13,15,16], supporting the clinical relevance of Ropeg-IFNa as a stabilizing or cytoreducing option under routine-care conditions. However, because most patients were pretreated and given the absence of a control arm, treatment effects should be interpreted cautiously as improvements over antecedent therapy rather than as causal effects relating solely to Ropeg-IFNa.

Molecular findings showed a trend in decreasing JAK2 V617F VAF in our real-world cohort, consistent with trial experience. In PROUD-PV, JAK2 V617F VAF fell from 41.9% to 30.7% at 12 months (−26.7% relative VAF), and in CONTINUATION-PV by >50% at 36 months [9]. Median VAF in our cohort significantly declined over a 14.8-month median follow-up, reflecting these early dynamics. Notably, sequential VAF decreases were observed beyond PV also in pre-PMF and MPN-U, suggesting interferon-driven clonal reduction is not restricted to PV. Although we lacked longitudinal on-treatment VAF for ET, early SURPASS-ET data similarly indicate JAK2 V617F VAF declines (33.7% to 25.3% at 12 months) [19]. In line with recent exposure–response analyses suggesting that higher Ropeg-IFNa exposure is associated with greater JAK2 V617F allele burden reduction [25], JAK2 V617F VAF measurements in a subset of patients with serial measurements in our real-world cohort also showed a respective trend without reaching statistical significance. Limited serial data were available for non-*JAK2* mutations, and the impact of Ropeg-IFNa on this broader clonal architecture warrants further study.

By subtype, hematologic gains were more evident in non-PV patients via improved thrombocytosis/leukocytosis control (Figure 2), whereas molecular reductions were predominantly documented in PV with confirmatory signals in one MPN-U.

A brief overlap of ruxolitinib and Ropeg-IFNa in one MPN-U patient illustrates a potentially complementary strategy: rapid symptomatic and splenic control from JAK2 inhibition plus interferon’s disease-modifying effect. Combination approaches have yielded encouraging signals, e.g., ruxolitinib + peginterferon alfa-2a in newly diagnosed PV yielded 60% molecular remissions with MPN10 improvements [26], and a prospective trial of Ropeg-IFNa plus JAK2 inhibition is ongoing (NCT06770842).

Tolerability was acceptable overall. However, 3/20 (15%) discontinued Ropeg-IFNa because of adverse events, predominantly mood disturbances (anxiety/depression). This rate is higher than in several real-world series: Huang et al. reported no toxicity-related discontinuations (median 12.4 months) [13]. Palandri et al. noted continued therapy in all patients with mainly grade 1–2 flu-like symptoms/alopecia (mean ~22 months) [16]. Popova et al. observed side effects in ~6% without discontinuations (median 24 months) [15], and Okikiolu et al. likewise reported no toxicity-driven stops (~11.6 months) [14]. It also exceeds the ~8% treatment-related discontinuation over five years in PROUD-PV/CONTINUATION-PV, where anxiety and depression were each reported once [9]. Our higher adverse event frequency likely reflects a high awareness and active capture of treatment-emergent symptoms (including grade 1 flu-like events). Given the overall limited number of patients, relative proportions should be interpreted with a certain caution. Notably, one of the three mood-related treatment discontinuations occurred in a patient with previous interferon-associated depression reflecting an increased risk. Overall, these findings underscore the need for careful psychiatric history, counseling, and active monitoring. In our cohort, mood symptoms were captured by patient report and clinical follow-up without structured screening. Importantly, Ropeg-IFNa is contraindicated in severe psychiatric illness per regulatory guidance [27,28] and these findings are in accordance with the official warnings for IFNa therapy. Increased awareness and regular monitoring may help mitigate this risk in future practice.

Limitations of our study include retrospective design, limited sample size, individualized dosing and titration, heterogeneity of previous therapies, as well as in intervals of laboratory measurements and symptom follow-up, and incomplete capture of some parameters (e.g., phlebotomy frequency, spleen size). These factors reflect real-world practices but introduce variability. Nonetheless, to our knowledge, this study is the largest single-center real-world series spanning multiple MPN subtypes and prior therapies, extending evidence beyond trial populations.

In summary, Ropeg-IFNa produced favorable hematologic and early molecular effects in a heterogeneous, pretreated MPN cohort. While overall tolerability was acceptable, mood-related discontinuations merit vigilance. Notably, we provide initial real-world signals of efficacy and tolerability in MPN-U. These findings support Ropeg-IFNa use across MPN subtypes in practice and highlight the value of tracking JAK2 V617F burden alongside proactive tolerability monitoring to optimize outcomes in real-world MPN patients.

## Figures and Tables

**Table 1:** Demographic, laboratory, and clinical parameters of MPN patients before initiation of Ropeg-IFNa treatment (=at baseline).

Variable	All Patients (n = 20)	PV (n = 11)	ET (n = 3)	Pre-PMF (n = 2)	MPN-U (n = 4)
Age, median (range)	52.9 (34–77)	51.9 (34–77)	57 (38–70)	49 (43–55)	54.5 (38–70)
Sex, n (M/F)	11/9	7/4	2/1	1/1	1/3
Disease duration, (months) median (range)	21.5 (2–300)	35 (6–300)	5 (4–32)	3 (2–4)	60 (6–96)
Hematocrit (%), mean ± SD	41.4 ± 4.6	42.0 ± 4.5	42.3 ± 5.5	37.5 ± 6.4	41.0 ± 4.3
Hemoglobin (g/L), mean ± SD	138.0 ± 13.2	140.0 ± 10.4	143.0 ± 20.6	122.5 ± 13.4	142.7 ± 16.4
Leukocyte count (G/L), mean ± SD	7.2 ± 3.4	5.9 ± 2.6	7.7 ± 1.0	9.8 ± 4.5	9.0 ± 6.1
Platelet count (G/L), mean ± SD	417.6 ± 287.6	385.1 ± 371.9	489.3 ± 85.2	524.0 ± 223.4	383.0 ± 149.8
JAK2 V617F VAF analysis performed, n (%)	13 (65)	7 (64)	2 (67)	2 (100)	2 (50)
JAK2 V617F VAF %, median (range)	12.0 (1.4–74.6)	21.2 (1.4–74.6)	9.1 (6.3–12.0)	37.0 (10.8–63.2)	12.4 (10.9–14.0)
Additional mutations in NGS (positive/tested)	5/9	ASXL1, DNMT3A (2/4)	EZH2, ASXL1 (1/2)	None (0/1)	BCOR, TET2, NF1 (2/2)
MPN10 SAF analysis performed, n (%)	12 (55)	6 (55)	2 (67)	1 (50)	3 (75)
MPN10 SAF median (range)	5.5 (0–36)	5.0 (3–15)	18 (0–36)	0	6 (5–24)
ELN thrombotic risk score (high vs. low)	11/9	3/8	3/0	1/1	4/0
Patients with previous thromboembolism (n, %)	10 (50)	2 (18)	3 (100)	1 (50)	4 (100)
Previous cytoreductive therapy (n, %) *	18 (90)	10 (91)	3 (100)	2 (100)	3 (75)
Previous phlebotomy (n, %)	9 (45)	8 (73)	0 (0)	0 (0)	1 (25)
Previous hydroxyurea (n, %)	13 (65)	6 (55)	3 (100)	2 (100)	2 (50)
Previous pegylated interferon alfa-2a (n, %)	8 (40)	8 (73)	0 (0)	0 (0)	0 (0)
Previous JAK2 inhibition (n, %)	2 (10)	1 (9)	0 (0)	0 (0)	1 (25)
Previous anticoagulation (n, %)	10 (50)	3 (27)	3 (100)	0 (0)	4 (100)
Previous antiaggregation (n, %)	8 (40)	6 (55)	0 (0)	2 (100)	0 (0)
Ropeg-IFNa starting dose (µg Q2W, mean ± SD)	115.7 ± 78.7	155.7 ± 85.7	66.7 ± 28.9	75 ± 35.4	62.5 ± 25

* previous cytoreductive therapy included hydroxyurea, pegylated interferon alfa-2a, or JAK2 inhibition with ruxolitinib.

## Data Availability

The patient data that support the findings of this study are not openly available due to reasons of sensitivity and are available from the corresponding author upon reasonable request. Data are located in controlled access data storage at Inselspital Bern.

## Abbreviations

The following abbreviations are used in this manuscript:

CTCAE	Common Terminology Criteria for Adverse Events
ET	Essential Thrombocythemia
IFNa	Interferon-Alpha
MPN	Myeloproliferative Neoplasm
MPN-U	MPN unclassifiable
Pre-PMF	Prefibrotic Primary Myelofibrosis
PV	Polycythemia Vera
Ropeg-IFNa	Ropeginterferon alfa-2b
VAF	Variant Allele Frequency
